# Poly(ADP-ribose) Polymerase 1 Is Indispensable for Transforming Growth Factor-β Induced Smad3 Activation in Vascular Smooth Muscle Cell

**DOI:** 10.1371/journal.pone.0027123

**Published:** 2011-10-31

**Authors:** Dan Huang, Yan Wang, Lin Wang, Fengxiao Zhang, Shan Deng, Rui Wang, Yun Zhang, Kai Huang

**Affiliations:** 1 Department of Cardiovascular Diseases, Union Hospital, Tongji Medical College, Huazhong University of Science and Technology, Wuhan, China; 2 Central Laboratory, Union Hospital, Tongji Medical College, Huazhong University of Science and Technology, Wuhan, China; 3 Key Laboratory of Cardiovascular Remodeling and Function Research, Qilu Hospital, Shandong University, Jinan, China; University of Hong Kong, Hong Kong

## Abstract

**Background:**

Transforming growth factor type-β (TGF-β)/Smad pathway plays an essential role in vascular fibrosis. Reactive oxygen species (ROS) generation also mediates TGF-β signaling-induced vascular fibrosis, suggesting that some sort of interaction exists between Smad and redox pathways. However, the underlying molecular mechanism is largely unknown. This study aims to investigate the influence of poly(ADP-ribose) polymerase 1 (PARP1), a downstream effector of ROS, on TGF-β signaling transduction through Smad3 pathway in rat vascular smooth muscle cells (VSMCs).

**Methods and Results:**

TGF-β1 treatment promoted PARP1 activation through induction of ROS generation in rat VSMCs. TGF-β1-induced phosphorylation and nuclear accumulation of Smad3 was prevented by treatment of cells with PARP inhibitor, 3-aminobenzamide (3AB) or N-(6-oxo-5,6-dihydrophenanthridin-2-yl)-2-(N,N-dimethylamino)acetami (PJ34), or PARP1 siRNA. TGF-β1 treatment promoted poly(ADP-ribosy)lation of Smad3 via activation of PARP1 in the nucleus. Poly(ADP-ribosy)lation enhanced Smad-Smad binding element (SBE) complex formation in nuclear extracts and increased DNA binding activity of Smad3. Pretreatment with 3AB, PJ34, or PARP1 siRNA prevented TGF-β1-induced Smad3 transactivation and expression of Smad3 target genes, including collagen Iα1, collagen IIIα1 and tissue inhibitor of metalloproteinase 1, in rat VSMCs.

**Conclusions:**

PARP1 is indispensable for TGF-β1 induced Smad3 activation in rat VSMCs. Targeting PARP1 may be a promising therapeutic approach against vascular diseases induced by dysregulation of TGF-β/Smad3 pathway.

## Introduction

Transforming growth factor type-β (TGF-β)/Smad pathway is critical for maintaining normal vascular structure. It regulates growth, differentiation, migration and proliferation of vascular cells [1]–[4]. Dysregulation of TGF-β/Smad pathway may lead to vascular fibrosis [1]–[6]. In TGF-β/Smad pathway, activated receptor-regulated Smads (RSmads), Smad2 and Smad3, mediate TGF-β signaling from cell membrane to the nucleus to regulate target gene transcription [7]. Besides RSmads, reactive oxygen species (ROS) also mediate TGF-β signaling transduction; and inhibition of ROS generation effectively prevents TGF-β1-induced over-production of collagens and tissue fibrosis [6], [8], [9]. These findings suggest that some sort of interaction exists between Smad and redox pathways in TGF-β signaling transduction. However, it remains elusive what factor mediates the interaction.

Poly(ADP-ribose)polymerase-1 (PARP1), an abundant and ubiquitous nuclear enzyme present in eukaryotes, accounts for about 90% of total cellular PARP activity. In the nucleus, activated PARP1 participates in a set of cellular processes by catalyzing the transfer of ADP-ribose from nicotinamide adenine dinucleotide (NAD^+^) onto nuclear acceptor proteins, including transcription factors and itself [10]–[12]. PARP1 can be activated by DNA strand breaks due to ROS [12], [13]. Recently, studies showed that knockout of PARP1 or suppression of PARP activity by PARP inhibitor prevented excessive production of collagens and development of tissue fibrosis under pathological conditions [10], [14]–[16]. However, the underlying mechanism remains elusive.

Vascular smooth muscle cell (VSMC) is the major cellular component of blood vessel wall and the major producer of vascular extracellular matrix (ECM) [17]. We examined the molecular link and interaction between PARP1 and Smad pathway in TGF-β-induced mRNA expression of ECM remodeling-related genes in rat VSMCs. We showed that treatment with antioxidant prevented TGF-β1-induced PARP1 activation. Knockdown of PARP1 or inhibition of PARP activity prevented TGF-β1-induced expression of Smad3 target genes, including collagen I (CoI) α1, CoIIIα1 and tissue inhibitor of metalloproteinase 1 (TIMP1). Activation of PARP1 was involved in the regulation of nuclear accumulation, sequence specific DNA binding and transactivation of Smad3 in TGF-β1-treated cells.

## Results

### 1. ROS mediated TGF-β1-induced PARP1 activation in VSMCs

**Figure 1:**
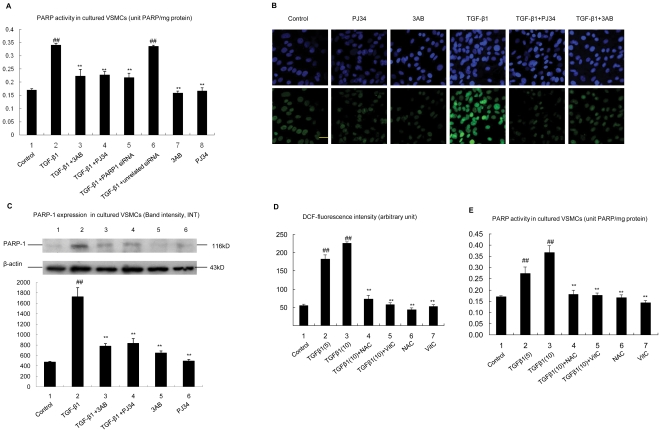
ROS mediated TGF-β1-induced PARP1 activation in VSMCs. A. PARP activity was assessed in VSMCs. Cells were pretreated with vehicles (PBS), 3AB (10 mmol/L, 24 h) or PJ34 (10 µmol/L, 24 h), or transfected with PARP1 siRNA (50 nmol/L, 48 h) or unrelated siRNA (50 nmol/L, 48 h), followed by TGF-β1 (10 ng/ml, 2 h) or vehicles (PBS) treatment. The effect of PARP1 siRNA on PARP1 level was shown in Figure S1. B. Confocal immunofluorescence assay was used to detect the poly(ADP-ribosy)lated protein expression (green fluorescence) in VSMCs (scale bar = 100 µm). Cells were pretreated with vehicles (PBS), 3AB or PJ34, followed by TGF-β1 or vehicles treatment. Hoechest (blue fluorescence) was used to stain the cell nuclei. C. Western-blot assay was used to detect PARP1 expression in VSMCs pretreated with vehicles (PBS), 3AB or PJ34, followed by TGF-β1 or vehicles treatment. D. Intracellular ROS in VSMCs were assessed by DCFH2-DA. Cells were pretreated with vehicles (PBS), NAC (10 mmol/L, 24 h) or VitC (100 µmol/L, 24 h), followed by TGF-β1 (5, 10 ng/ml, 2 h) or vehicles (PBS) treatment. E. PARP activity was assessed in VSMCs treated as indicated. Data are expressed as the mean±S.E.M, n = 6, ## P<0.01 compared to the control group, ** P<0.01 compared to the TGF-β1 treatment group.

The influence of TGF-β1 on PARP1 activation in VSMCs was explored. TGF-β1 treatment increased the cellular PARP activity. Treatment with PARP inhibitor, 3-aminobenzamide (3AB) or N-(6-oxo-5,6-dihydrophenanthridin-2-yl)-2-(N,N-dimethylamino)acetamide (PJ34), or PARP1 siRNA prevented TGF-β1-induced increase in PARP activity of cultured rat VSMCs (Figure 1A). In line with this result, confocal immunofluoresence assay with anti-poly(ADP-ribose) polymer (PAR) antibody indicated that TGF-β1 treatment led to up-regulation of poly(ADP-ribosy)lated proteins in VSMCs. Inhibition of PARP activity by 3AB or PJ34 decreased TGF-β1-induced poly(ADP-ribosy)lation levels of proteins (Figure 1B). Activation of PARP1 increased expression of its own gene [18], [19]. In this study, treatment with 3AB or PJ34 prevented TGF-β1-induced increase in PARP1 expression (Figure 1C).

PARP1 can be activated by ROS [12], [15]. We demonstrated that TGF-β1 treatment promoted ROS generation in VSMCs (Figure 1D); and pretreatment of cells with antioxidant, vitamin C or N-acetylcysteine (NAC), inhibited TGF-β1-induced increase in PARP activity (Figure 1E). These results indicated that TGF-β1 activated PARP1 through induction of ROS generation.

### 2. Inhibition of PARP1 prevented TGF-β1-induced mRNA expression of Smad3 target genes

**Figure 2:**
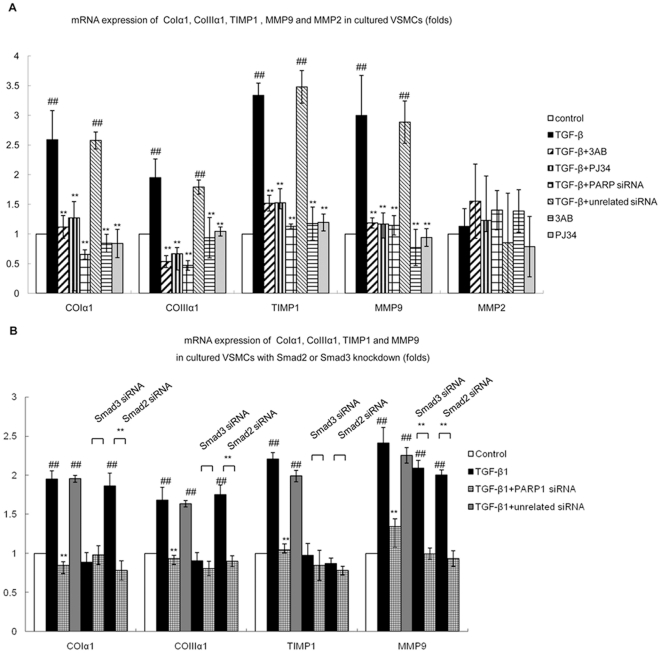
Inhibition of PARP1 prevented TGF-β1-induced gene transcription in VSMCs. A. Real-time PCR was used to detect mRNA expression of CoIα1, CoIIIα1, TIMP1, MMP9 and MMP2 in VSMCs. Cells were pretreated with vehicles (PBS), 3AB (10 mmol/L, 24 h) or PJ34 (10 µmol/L, 24 h), or transfected with PARP1 siRNA (50 nmol/L, 48 h) or unrelated siRNA (50 nmol/L, 48 h), followed by TGF-β1 (10 ng/ml, 2 h) or vehicles (PBS) treatment. B. mRNA expression of CoIα1, CoIIIα1, TIMP1 and MMP9 in Smad2-knockdown (50 nmol/L, 48 h) or Smad3-knockdown (50 nmol/L, 48 h) VSMCs. Cells were treated with PARP1 siRNA (50 nmol/L, 48 h) or unrelated siRNA (50 nmol/L, 48 h), followed by TGF-β1 (10 ng/ml, 2 h) treatment. The effects of PARP1, Smad2 or Smad3 siRNA on levels of PARP1, Smad2 or Smad3 were shown in Figure S1. Data are expressed as the mean±S.E.M., n = 6, ## P<0.01 compared to the control group, ** P<0.01 compared to the TGF-β1 treatment group.

TGF-β1 treatment resulted in up-regulation of mRNA levels of CoIα1, CoIIIα1, TIMP1, matrix metalloproteinase (MMP) 9, but not of MMP2, in VSMCs (Figure 2A). Knockdown of PARP1 by siRNA or pretreatment with 3AB or PJ34 inhibited TGF-β1-induced increase in mRNA expression of above mentioned genes (Figure 2A), indicating that PARP1 was involved in the regulation of TGF-β1-induced gene transcription.

Both Smad2 and Smad3 are downstream effectors of TGF-β signaling. Knockdown of Smad3 by siRNA prevented TGF-β1-induced transcription of CoIα1 and CoIIIα1 genes, while knockdown of Smad2 failed to inhibit TGF-β1-induced CoIα1 and CoIIIα1 transcription (Figure 2B), indicating that CoIα1 and CoIIIα1 were Smad3 target genes. Knockdown of either Smad3 or Smad2 by siRNA inhibited TGF-β1-induced gene transcription of TIMP1 (Figure 2B), indicating that TIMP1 was the target gene of both Smad2 and Smad3. However, TGF-β1-induced MMP9 gene transcription was not influenced by Smad2 siRNA or Smad3 siRNA in VSMCs (Figure 2B), suggesting that TGF-β1-induced MMP9 transcription was not mediated through Smad2/3 pathway. The transcription of CoIα1, CoIIIα1 and TIMP1 were influenced by several transcription factors. To explore whether the influences of PARP1 on TGF-β1-induced transcription of these genes were mediated through Smad3 pathway, both Smad3 and PARP1 were knocked-down in cells. Results showed that treatment with PARP1 siRNA failed to influence mRNA expression of Smad3 target genes in Smad3-knockdown cells treated with or without TGF-β1 (Figure 2B), implicating that the effects of PARP1 on TGF-β1-induced transcription of these genes were mediated through Smad3 pathway.

### 3. Smad3 were poly(ADP-ribosy)lated by PARP1 in VSMCs

**Figure 3:**
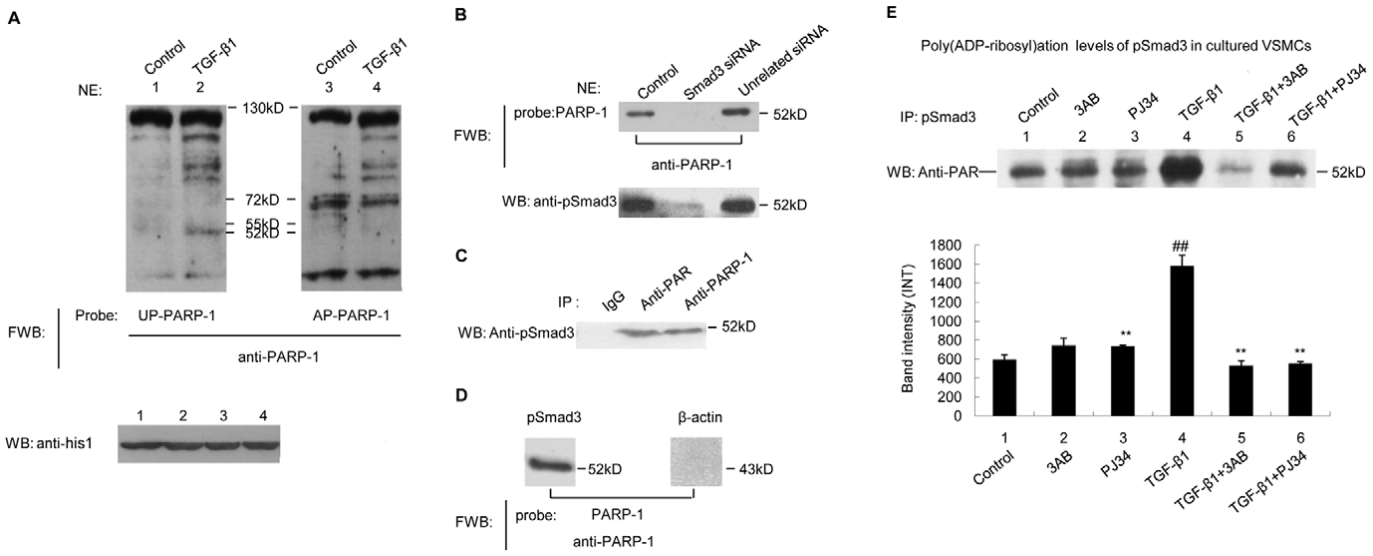
Phosphorylated Smad3 (pSmad3) was poly(ADP-ribosy)lated by PARP1. A. Far-western-blot assay of nuclear extracts from non-treated or TGF-β1 treated VSMCs. Un-poly(ADP-ribosy)lated PARP1 (UP-PARP1), auto-poly(ADP-ribosyl)ated PARP1 (AP-PARP1) and β-actin protein (served as negative control, data not shown) were used as probe, respectively. histone 1 served as loading control (lower panel). B. Far-western-blot assay was used to detect the binding of PARP1 to pSmad3 in VSMCs transfected with Smad3 or unrelated siRNA (50 nmol/L, 48 h). PARP1 was used as probe. The effects of Smad3 siRNA on Smad3 levels were shown in lower panel. C. Immunoprecipitation of PAR or PARP1-bound proteins from TGF-β1-treated VSMCs, followed by western-blot assay using anti-pSmad3 antibody. Unspecific IgG served as negative control. D. Far-western-blot assay was used to detect the binding of PARP1 to pSmad3 in cell free system. E. Immunoprecipitation of pSmad3 from VSMCs treated as indicated, followed by western-blot assay using anti-PAR antibody. Total Smad3 and histone 1 served as loading control (data not shown). FWB, far-Western-blot; IP, Immunoprecipitation; NE, nuclear extracts; WB, Western-blot. Data are expressed as the mean±S.E.M, n = 5 to 6. ## P<0.01 compared to the control group, ** P<0.01 compared to the TGF-β1 treatment group.

To explore the mechanism underlying the influence of PARP1 on Smad3 target gene transcription, we screened for interacting nuclear protein of PARP1 by far-western-blot assay using PARP1 protein as probe. Results showed that un-poly(ADP-ribosy)lated PARP1 (UP-PARP1) specifically bound to a 52 kD protein in nuclear extracts from TGF-β1-treated VSMCs, but not from non-treated cells (Figure 3A), indicating that the PARP1-bound 52 kD protein entered the nucleus after TGF-β1 stimulation. Since Smad3 was a 52 kD protein and entered the nucleus after phosphorylation [7], [20], this protein was speculated to be Smad3. In line with this speculation, UP-PARP1 failed to bind to any 52 kD nuclear protein in Smad3-knockdown VSMCs (Figure 3B). Far-western-blot assay using phosphorylated Smad3 (pSmad3) protein as probe also showed that pSmad3 could specifically bind to PARP1 in nuclear extracts (Figure S2A). Thereafter, nuclear extracts from TGF-β1-treated cells were subjected to co-immunoprecipitation with antibody specific for PARP1 and pSmad3 respectively. Western-blot assay revealed that pSmad3 protein was co-immunoprecipitated with anti-PARP1 antibody (Figure 3C), and *vice versa* (Figure S2B). In cell-free system, we also found that pSmad3 could specifically bind to recombinant PARP1 (Figure 3D), and *vice versa* (Figure S2C).

Whether pSmad3 could be poly(ADP-ribosy)lated by PARP1 in VSMCs was investigated. TGF-β1 treatment promoted poly(ADP-ribosy)tion of a 52 kD nuclear protein; and knockdown of PARP1 by siRNA prevented TGF-β1-induced poly(ADP-ribosy)lation of this protein (Figure S2D). The results indicated that a 52 kD nuclear protein was poly(ADP-ribosy) lated by PARP1 in TGF-β1-treated cells. Nuclear extracts were thereafter subjected to immunoprecipitation with antibody specific for PAR. Western-blot assay showed that pSmad3 was immunoprecipitated with anti-PAR antibody (Figure 3C). Immunoprecipitation assay with antibody specific for pSmad3 was then carried out. Western-blot assay with anti-PAR antibody revealed that TGF-β1 treatment increased the amount of eluted poly(ADP-ribosy)lated pSmad3; and treatment with 3AB or PJ34 prevented TGF-β1-induced poly(ADP-ribosy)lation of pSmad3 (Figure 3E). In cell free system, incubation of pSmad3 with active DNA, NAD^+^ and PARP1 led to poly(ADP-ribosyl)ation of pSmad3 (Figure S2E). These results suggested that TGF-β1 treatment promoted poly(ADP-ribosy)lation of pSmad3 through activation of PARP1.

### 4. Poly(ADP-ribosy)lation increased the DNA binding of Smad3

**Figure 4:**
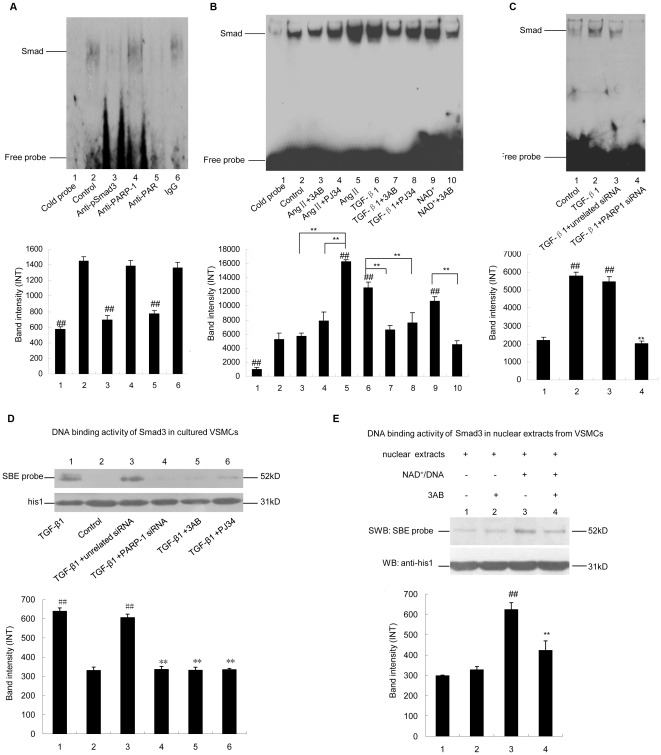
Poly(ADP-ribosy)lation increased DNA binding of Smad3. A. Supershift assay of nuclear extracts from TGF-β1-treated VSMCs using SBE as probe. Lane 2 served as control. Lane 3–6 indicated incubation of nuclear extracts with anti-pSmad3 antibody, anti-PARP1 antibody, anti-PAR antibody or unspecific IgG (negative control). B. EMSA assay of nuclear extracts from VSMCs using SBE as probe. Cells were pretreated with vehicles (PBS), 3AB (10 mmol/L, 24 h) or PJ34 (10 µmol/L, 24 h), followed by TGF-β1 (10 ng/ml, 2 h), AngII (0.1 µmol/L, 24 h) or vehicles (PBS) treatment. Lane 9–10 indicated incubation of nuclear extracts from non-treated cells with 10 µM NAD^+^ in the absence or presence of 10 mM 3AB. C. EMSA assay of nuclear extracts from VSMCs using SBE as probe. Cells were transfected with PAPR1 siRNA (50 nmol/L, 48 h) or unrelated siRNA, followed by TGF-β1 (10 ng/ml, 2 h) treatment. The effects of PARP1 siRNA on PARP1 levels were shown in Figure S1. D. Southwestern-blot assay was used to detect SBE binding of Smad3. VSMCs were treated as indicated. E. Nuclear extracts from non-treated cells were incubated with 3AB, NAD^+^/active DNA, or NAD^+^/active DNA/3AB, Southwestern-blot assay was used to detect SBE binding of Smad3. Data are expressed as the mean±S.E.M, n = 6. ## P<0.01 compared to the control group, ** P<0.01 compared to the AngII treatment group, or the TGF-β1 treatment group, or the NAD^+^ /active DNA treatment group.

Smad3 can directly bind to Smad-binding element (SBE) in promoter of target gene. Smad2 participates in DNA-bound complexes via its interaction with Smad4 [7], [20]. The roles of Smad2 and Smad3 in TGF-β1-induced Smad-SBE complex formation were explored, respectively. EMSA assay showed that knock down of Smad2 failed to influnce TGF-β1-induced Smad-SBE complex formation, while knock down of Smad3 abrogated the band of Smad-SBE complex (Figure S3A), suggesting that Smad3, but not Smad2, was the major component of Smad-SBE complex. In line with this finding, supershift assay showed that incubation of nuclear extracts from TGF-β1-treated VSMCs with anti-pSmad3 antibody abrogated the band of Smad-SBE complex (Figure 4A). Interestingly, supershift assay also showed that incubation of anti-Smad2 or anti-Smad4 antibody with nuclear extracts from TGF-β1-treated cells failed to abrogate or shift the band of Smad-SBE complex, although the band intensity of Smad-SBE complex was slightly reduced when more anti-Smad2 or anti-Smad4 antibody was used (data not shown). All the data illustrated that Smad3 was a predominant component of Smad-SBE complex.

The influence of PARP1 on Smad-SBE complex formation was then investigated. Knockdown of PARP1 by siRNA, or inhibition of PARP activity by 3AB or PJ34 prevented TGF-β1-induced Smad-SBE complex formation in nuclear extracts (Figure 4B, C), suggesting that activation of PARP1 promoted Smad-SBE complex formation. Thereby, the direct effect of poly(ADP-ribosy)lation on Smad-SBE complex formation was investigated. Incubation of nuclear extracts from non-treated cells with NAD^+^ and SBE increased Smad-SBE complex formation; and this increases were inhibited by co-incubation with 3AB (Figure 4B). These results suggested that PARP1 promoted Smad-SBE complex formation through poly(ADP- ribosy)lation of nuclear proteins.

The effect of poly(ADP-ribosy)lation on Smad3 DNA binding was explored. With southwestern-blot assay, we demonstrated that knockdown of Smad3 by siRNA abrogated binding of SBE to a 52 kD nuclear protein, indicating that the SBE-bound 52 kD protein was Smad3 (Figure S3B). Moreover, southwestern-blot assay also showed that treatment with TGF-β1 increased the DNA binding of Smad3; and the TGF-β1-induced increase was prevented when PARP1 was knocked-down by siRNA or inhibited by 3AB or PJ34 (Figure 4D). Moreover, incubation of nuclear extracts from non-treated cells with NAD^+^ and active DNA also increased Smad3 DNA binding activity; and the increase was inhibited by co-incubation with 3AB (Figure 4E). Similar results were found in cell free experiment (Figure S3C). All the data indicated that poly(ADP-ribosy)lation increased DNA binding of Smad3.

Poly(ADP-ribosy)lation of PARP1 prevented its binding to DNA. Smad3 could bind to UP-PARP1, but not AP-PARP1, suggesting that PARP1 was not involved in Smad-SBE complex formation. In line with this finding, incubation of nuclear extracts with anti-PARP1 antibody failed to shift or abrogate the band of Smad-SBE complex (Figure 4A). Moreover, incubation of PARP1 protein with nuclear extracts did not influence Smad3-SBE complex formation (Figure S3D).

### 5. Inhibition of PARP1 prevented TGF-β1-induced nuclear accumulation of Smad3

**Figure 5:**
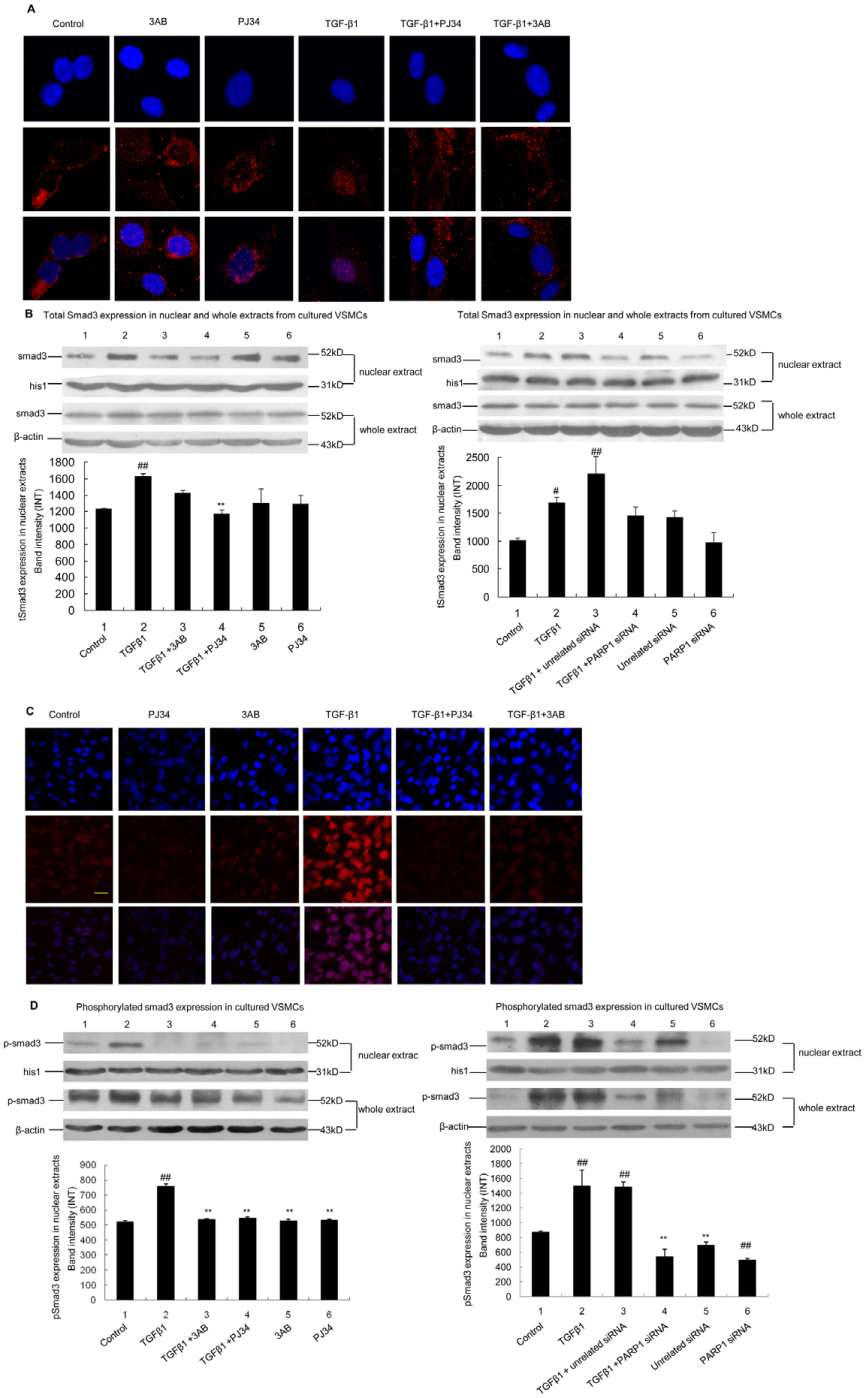
Inhibition of PARP1 prevented TGF-β1-induced nuclear accumulation of Smad3. A and C. Confocal immuno fluorescence assay was used to detect the expression of total Smad3 (A) and pSmad3 (C) (red fluorescence), respectively. Hoechest (blue fluorescence) was used to stain the cell nuclei (scale bar = 40 µm). Cells were pretreated with vehicles (PBS), 3AB (10 mmol/L, 24 h) or PJ34 (10 µmol/L, 24 h), followed by TGF-β1 (10 ng/ml, 2 h) or vehicles (PBS) treatment. B and D. Western-blot assay was used to detect the expression of total Smad3 (B) and pSmad3 (D) in VSMCs treated as indicated. Cells were pretreated with vehicles (PBS), 3AB (10 mmol/L, 24 h) or PJ34 (10 µmol/L, 24 h), or transfected with PARP1 siRNA (50 nmol/L, 48 h) or unrelated siRNA (50 nmol/L, 48 h), followed by TGF-β1 (10 ng/ml, 2 h) or vehicles (PBS) treatment. The effects of PARP1 siRNA on level of PARP1 was shown in Figure S1. Data are expressed as the mean S.E.M., n = 3. ## P<0.01 compared to the control group, ** P<0.01 compared to the TGF-β1 treatment group.

The influence of PARP1 on nuclear accumulation of total Smad3 (tSmad3) was explored. Confocal immunofluoresence assay with anti-Smad3 antibody showed that TGF-β1 treatment resulted in increased nuclear tSmad3 levels; and pretreatment with 3AB or PJ34 prevented the TGF-β1-induced increase (Figure 5A). Western-blot assay showed that TGF-β1 treatment increased tSmad3 levels in nuclear extracts, but not in whole extracts. Moreover, knockdown of PARP1 by siRNA or inhibition of PARP activity by 3AB or PJ34 prevented TGF-β1-induced increases in nuclear tSmad3 level, but did not affected tSmad3 level in whole extracts (Figure 5B).

**Figure 6:**
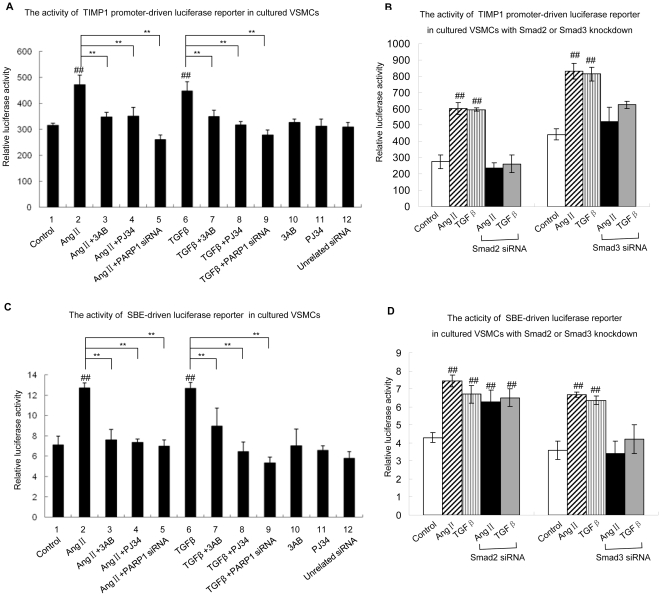
Inhibition of PARP1 prevented Smad3 transactivation. A and C. Luciferase activity assay was used to detect TIMP1 promoter-driven luciferase reporter activity (A) or SBE-driven luciferase reporter activity (C) in VSMCs, respectively. Cells were pretreated with vehicles (PBS), 3AB (10 mmol/L, 24 h) or PJ34 (10 µmol/L, 24 h), or transfected with PARP1 siRNA (50 nmol/L, 48 h) or unrelated siRNA (50 nmol/L, 48 h), followed by TGF-β1 (10 ng/ml, 2 h), AngII (0.1 µmol/L, 2 h) or vehicles (PBS) treatment. B and D. Luciferase activity assay was used to detect TIMP1 promoter-driven luciferase reporter activity (B) or SBE-driven luciferase reporter activity (D) in VSMCs. Cells were treated with vehicles (PBS), AngII (0.1 µmol/L, 2 h), TGF-β1 (10 ng/ml, 2 h), or transfected with Smad2 siRNA (50 nmol/L, 48 h) or Smad3 siRNA (50 nmol/L, 48 h) followed by AngII (0.1 µmol/L, 2 h) or TGF-β1 (10 ng/ml, 2 h) treatment. The effects of Smad2 or Smad3 siRNA on levels of Smad2 or Smad3 were shown in Figure S1. Data are expressed as the mean ± S.E.M., n = 6, # P<0.05, ## P<0.01 compared to the control group, ** P<0.01 compared to the AngII treatment group or the TGF-β1 treatment group.

Following TGF-β stimulation, Smad3 becomes phosphorylated at carboxyl terminal serine residues (Ser423 and 425) by TGF-β receptor I (TβRI) and then, enters the nucleus [7], [21]. We thereby investigated the influence of PARP1 on TGF-β1-induced Smad3 phosphorylation. Confocal immunofluoresence assay with anti-pSmad3 antibody (used to detect levels of Smad3 when phosphorylated at Ser423/425) showed that TGF-β1 treatment increased pSmad3 levels in VSMCs, especially in the nucleus; and pretreatment with 3AB or PJ34 prevented the TGF-β1-induced increase (Figure 5C). Western-blot assay also showed that TGF-β1 treatment increased pSmad3 levels in whole and nuclear extracts; and knockdown of PARP1 by siRNA or inhibition of PARP activity by 3AB or PJ34 prevented TGF-β1-induced pSmad3 increase in both whole and nuclear extracts (Figure 6D). These results indicated that activation of PARP1 promoted Smad3 phosphorylation in TGF-β1-treated cells.

### 6. Knockdown of PARP1 prevented Smad3 transactivation

To explore the direct influence of PARP1 on Smad3 target gene transcription, TIMP1 promoter (from +866bp to +1523bp) driven luciferase reporter was constructed and transfected into VSMCs[22]. Treatment with AngII or TGF-β1 increased luciferase reporter activity in VSMCs, and knockdown of PARP1 by siRNA or inhibition of PARP activity by 3AB or PJ34 prevented the AngII or TGF-β1-induced increase (Figure 6A), indicating that PARP1 contributed importantly to AngII or TGF-β1-induced TIMP1 promoter-driven luciferase reporter expression. Knockdown of either Smad2 or Smad3 prevented AngII or TGF-β1-induced increase in luciferase reporter activity (Figure 6B).

To explore the direct influence of PARP1 on Smad3 transactivation, SBE-driven luciferase reporter was constructed and transfected into VSMCs. Treatment with AngII or TGF-β1 increased luciferase reporter activity in VSMCs; and knockdown of PARP1 by siRNA or inhibition of PARP activity by 3AB or PJ34 prevented the TGF-β1 or AngII-induced increase (Figure 6C). These results indicated that activation of PARP1 mediated AngII or TGF-β1-induced Smad transactivation. To explore whether Smad2 or Smad3 mediated AngII or TGF-β1-induced SBE-driven reporter expression, they were knocked down respectively. Results showed that AngII or TGF-β1 treatment increased luciferase reporter activity in Smad2-knockdown, but not Smad3-knockdown, VSMCs. It suggested that activation of Smad3, but not of Smad2, enhanced the expression of SBE-driven luciferase reporter gene (Figure 6D). These results also illustrated that activation of PARP1 was critical for TGF-β1-induced Smad3 transactivation in VSMCs.

## Discussion

Transforming growth factor-β (TGF-β)/Smad pathway is an evolutionarily conserved signaling transduction pathway. Here, we present the evidence that PARP1 plays a critical role in TGF-β/Smad3 pathway activation. We demonstrated that activation of PARP1 was required for TGF-β1-induced Smad3 phosphorylation and nuclear accumulation. Poly(ADP-ribosy)lation of Smad3 by PARP1 increased its DNA binding activity and promoted Smad-SBE complex formation in the nucleus. Knockdown of PARP1 prevented Smad3 transactivation and target gene transcription.

TGF-β1 stimulation enhances the ROS generation through activation of NADPH oxidase in cells [6], [8], [23]-[25]. PARP1 is a downstream effector of NADPH oxidase and ROS [26]. We showed that TGF-β1 treatment increased PARP activity; and knockdown of PARP1 prevented TGF-β1-induced increase in PARP activity. This result is consistent with Peter Lonn's finding [27]. Under basic condition, PARP1 is a transcriptional repressor of its own gene since it directly binds to the certain secondary structures or scaffold/matrix-attachment regions in the promoter to prevent gene transcription [18], [19]. Auto-poly(ADP-ribosy)lation prevents PARP1 binding to the promoter of its own gene and thereby increases the transcription of itself [18], [19]. Thereby, ROS induced auto-modification of PARP1 enhances PARP1 gene transcription. In line with these findings, we showed that TGF-β1 treatment increased PARP1 expression, and pharmacological inhibition of PARP activation prevented the TGF-β1 induced up-regulation of PARP1 expression.

Smad2 and Smad3 are predominantly localized in the cytoplasm under basic condition [7]. After TGF-β stimulation, phosphorylated Smad2 or Smad3 forms homomeric or heteromeric complexes with Smad4, and enters the nucleus to regulate gene transcription [7], [20]. Our results suggested that PARP1 interacts with Smad3 in nucleus of rat VSMCs under basic conditions. When PARP1 is activated by TGF-β, it poly(ADP-ribosy)lates Smad3 and itself thereafter. After poly(ADP-ribosy)lation reaction, PARP1-Smad3 complex was dissociated and Smad3 bound to the promoter of its target gene. Although Smad4 could also be poly(ADP-ribosy)lated by PARP1 (Information S1), the mechanism underlying poly(ADP-ribosy)lation of Smad4 was different from that of Smad3. Smad4 distributes between nucleus and cytoplasm under basic condition [28]. We showed that Smad4 could just bind to AP-PARP1, but not UP-PARP1 (Information S1), indicating that PARP1 did not interact with Smad4 in cells under basic condition. These findings also suggested that Smad4 might be poly(ADP-ribosy)lated when PARP1 was activated in the absence of TGF-β stimulation.

Poly(ADP-ribosy)lation is an important post-transcriptional modification of proteins. It may enhance or inhibit the DNA binding of different nuclear proteins by changing their respective structure. For example, the DNA binding activity of PPAR-γ decreased dramatically after poly(ADP-ribosyl)ation [11], [12]; on the contrary, poly(ADP-ribosy)lation enhanced the DNA binding of AP-1 and NFAT [10], [29]. In this study, poly(ADP-ribosy)lation promoted Smad-SBE complex formation in nuclear extracts and increased DNA binding activity of pSmad3. Moreover, knockdown or inhibition of PARP1 prevented TGF-β1-induced increase in DNA binding of pSmad3. These results suggest a previously unappreciated possibility that TGF-β1 treatment may increase the DNA binding of pSmad3 through poly(ADP-ribosy)lation. Smad4 may form heteromeric complex with pSmad3 to regulate target gene transcription. DNA binding activity of Smad4 was also increased after poly(ADP-ribosy)lation, suggesting that poly(ADP-ribosy)lation of Smad4 might also contribute to TGF-β-induced Smad-SBE complex formation.

Nuclear accumulation of RSmads is a crucial step in TGF-β signaling transduction [6], [30].We showed that activation of PARP1 was required for TGF-β1-induced tSmad3 nuclear accumulation. Under basic condition, Smad3 translocates into the nucleus through direct association with nuclear import factor importin-β1 and FG-repeat containing nucleoporins such as CAN/Nup214 [31], [32]. After TGF-β stimulation, phosphorylation of Smad3 increases its nuclear accumulation by destabilizing its interaction with cytoplasmic retention factor Smad anchor for receptor activation (SARA) and enhancing its association with importin-β1 [31], [32]. Dephosphorylation of Smad2/3 by magnesium-dependent protein phosphatase 1alpha (PPM1A) promoted Smad2/3 nuclear export through induction of Smad2/3 binding to Ran-binding protein 3 (RANBP3) and exportin 4 [7], [33], [34]. These findings indicate that nuclear accumulation of Smad3 is phosphorylation dependent [20]. Knockdown or inhibition of PARP1 prevented TGF-β1-induced increase in pSmad3 in nuclear and whole extracts, indicating that activation of PARP1 is required for TGF-β1-induced phosphorylation of Smad3. These results suggest that PARP1 promotes tSmad3 nuclear accumulation by increasing its phosphorylation. It has been demonstrated that phosphorylation levels of Smad3 are regulated by TβRI, TβRII and PPM1A. Interestingly, knockdown or inhibition of PARP-1 did not affect the expression of these 3 enzymes in resting and TGF-β1-treated cells (data not shown). In addition to phosphorylation, interaction between Smad3 and transcriptional co-activator with PDZ-binding motif (TAZ) may also affect Smad3 nuclear accumulation [35], [36]. However, it is not clear whether PARP-1 affects the interaction between TAZ and Smad3. Moreover, the influences of PARP1 on PPM1A, RANBP3 and exportin 4 remained elusive. Thereby, it is necessary to clarify the detailed mechanism involved in the regulation of Smad3 phosphorylation and nuclear accumulation by PARP-1 in VSMCs.

Activation of Smad3, but not of Smad2, mediated TGF-β1-induced increase in SBE-driven luciferase activity and transcription of CoIα1 and CoIIIα1 genes in VSMCs. Inhibition of PARP1 prevented TGF-β1-induced expression of SBE-driven luciferase reporter and Smad3 target genes, indicating that activation of PARP1 was required for TGF-β1-induced Smad3 transactivation. Besides Smad3 pathway, TGF-β stimulation also leads to ROS generation through activation of NADPH oxidase [6], [8], [37], [38]. We found that treatment with antioxidant vitamin C or NAC prevented TGF-β-induced mRNA expression of Smad3 target genes, CoIα1 and CoIIIα1, in VSMCs (data not shown). Since activation of PARP1, a downstream effector of ROS, is required for TGF-β1-induced Smad3 transactivation and target gene expression, it is suggested that PARP1 mediates TGF-β1-induced interaction between redox and Smad3 pathways in VSMCs.

In summary, the data reveal a novel mechanism underlying activation of TGF-β/Smad3 signaling pathway in VSMCs: 1. TGF-β stimulation activates PARP1; 2. activation of PARP1 promotes Smad3 phosphorylation and nuclear accumulation; 3. PARP1 binds to and poly(ADP-ribosy)lates pSmad3 in nucleus; 4. poly(ADP-ribosy)lation promotes binding of Smad3 to its consensus DNA binding element in the promoter to regulate target gene transcription. This finding will help us explore the mechanism underlying involvement of PARP1 in the pathogenesis of vascular fibrosis. Moreover, it also suggests that PARP1 may be a promising therapeutic target against vascular fibrosis and other diseases induced by dysregulation of TGF-β-activated Smad3 signaling.

## Materials and Methods

### Ethics Statement

All animal work used in this study were conformed to the National Institutes of Health (NIH) Guide for the Care and Use of Laboratory Animals, and approved by the Ethics Committee of Tongji Medical College, Huazhong University of Science and Technology, China. The permit number of this study is “[2010]S058”.

### Cell Culture

Rat vascular smooth muscle cells (VSMCs) were isolated by enzymatic digestion of thoracic aortic media from male Sprague-Dawley rats (250–300g, obtained from Tongji Medical College, HUST) by the method of Michel [39]. Cells were maintained in DMEM with 20% FBS. The purity of the VSMCs preparation was evaluated by staining the cells with monoclonal antibodies to smooth muscle cell α-actin. More than 96% of cells were immunoreactive. Experiments were performed on cells at 3 to 10 passages after primary culture. In various experiments, supplements were used at the following concentrations: 10 mmol/L 3AB (Sigma), 10 µmol/L PJ34 (Alexis Biochemicals), 5 or 10 ng/ml TGF-β1 (PeproTech), 0.1 µmol/L AngII (Sigma), 10 mmol/L N-acetylcysteine (NAC, Beyotime, China), 100 µmol/L vitamin C (Sigma) or the relevant vehicle controls (PBS).

### RNA interference and transfection

Small interfering RNA (siRNAs) was synthesized by RiBoBio Co. Ltd (Guangzhou, China). The sequences of siRNAs were listed in Table S1. The cultured cells were transfected in 6-well plates at 70% confluence. Transfection of siRNA was performed at a final concentration of 50 nmol/L using Lipofectamine 2000 (Invitrogen).

### Evaluation of transfection efficiency

VSMCs were plated in 24-well plates 24 hour prior to transfection with 50 nmol/L Cy3-labeled siRNA as described above. Transfections were performed in triplicate for each treatment. One part was fixed for flow cytometric analyses as previously described [40]. And the others were washed with PBS, fixed in paraformaldehyde 4% for 30 min in the dark, washed with methanol for 3 to 5 times, and washed again with PBS. After several washes in PBS, the cover slips were mounted on PBS/glycerin. Cells were photographed under a light or fluorescence microscope (for Cy3, wavelength 555 nm; Olympus Microscope BX-51).

### Measurement of intracellular reactive oxygen species (ROS) levels

The level of intracellular ROS was determined on the basis of the oxidative conversion of cell permeable 20,70-dichloro fluorescein diacetate (DCFH-DA) to fluorescent dichloro fluorescein (DCF) upon reaction with hydroxyl radical, hydrogen peroxide, or peroxynitrite. Brie fly, cells were incubated with control media (PBS) or TGF-β1 in the presence or absence of antioxidants (NAC or vitamin C) for 2 h. Then cells were washed twice with cold PBS (pH 7.4) and incubated with DCFH-DA at room temperature for 30 min in dark. Fluorescent signal was recorded by using a fluorescence microscopy (488 nm filter; Olympus Microscope BX-51, Japan). The fluorescence intensity of eight fields per dish was measured and the reactive oxygen species (ROS) level was quantified by measurement of fluorescence intensity with HMIAS-2000 software. Three parallel experiments were performed. Results were shown as the mean value.

### Preparation of whole extracts and nuclear extracts

Whole cell extracts and nuclear extracts were prepared as described previously [10], [11]. Protein extracts were quantitated using the Bradford assay. The obtained extracts were stored at −80°C until use.

### PARP activity assay

PARP activity was assayed using the universal colorimetric PARP assay kit (Trevigen), based on the incorporation of biotinylated ADP-ribose onto histone proteins. Cell lysates containing 50 µg of protein were loaded into a 96-well plate coated with histones and biotinylated poly ADP-ribose, allowed to incubate for 1 hour, treated with strep-HRP, and read at 450 nm in a spectropehotometer.

### Western Blot

Equal protein amounts were loaded onto SDS-PAGE gels. After running gels, proteins were transferred onto nitrocellulose membranes. Membranes were blocked in 5% nonfat milk and primary antibody incubations were performed with 3% BSA (overnight at 4°C). Antibodies used were anti-PARP1 (R&D, 1∶1000), anti-PAR (Trevigen, 1∶1000), anti-hist1, anti-β-actin (Santa Cruz, 1∶1000), anti-Smad4, anti-Smad3, and anti-phosphorylated Smad3 (Cell Signaling Technology, 1∶800). Then membranes were incubated with peroxidase-conjugated secondary antibody at room temperature for 2 hours. Specific band was detected with chemiluminescence assay (ECL detection reagents, Pierce) and recorded on x-ray film. Quantity One software was used to quantify the intensities of bands.

### Real Time RT-RCR

RNA from cultured cells was isolated using Trizol reagent (Takara Biotechnology) according to the manufacturer's instruction. cDNA was synthesized using RNA PCR Kit (Takara Biotechnology) and used as PCR template. Quantitative PCR was performed on ABI PRISM 7900 Sequence Detector system (Applied Biosystems) using SYBR Green I Assay (Takara Biotechnology). GAPDH was used as endogenous control. Relative gene expression level (the amount of target, normalized to endogenous control gene) was calculated using the comparative Ct method formula 2^-ΔΔ^Ct. The sequences of primers for PCR were listed in Table S2.

### Confocal immunofluorescence

Cells seeded on glass chamber slides at a density of 2,000 cells per chamber were washed, fixed in ice-cold 4% paraformaldehyde for 30 min, and permeabilized in 100 mM phosphate buffer containing 0.2% TritonX-100 (Sigma-Aldrich) for 10 min. The cells were then incubated with 5% bovine serum albumin (BSA) and immunolabeled with the indicated antibody (anti-PAR antibody, anti-phosphorylated Smad3 antibody and anti-Smad3 antibody, respectively) at room temperature for 1 hour. After washing, the cells were incubated with Alexa Fluor 594 Donkey anti-rabbit IgG or Alexa Fluor 488 goat anti-mouse IgG (Molecular Probes, Eugene, OR) for 1 hour. After washing, Hoechst (1 mg/ µL; Sigma-Aldrich) was added to stain the cell nuclei on ice. Cell fluorescence was imaged on confocal microscope (A1si, Nikon).

### In vitro protein-protein interaction assay (far-Western blot)

Far-western blot assays were performed by resolving protein samples by SDS-PAGE and transferring them to polyvinylidenefluoride membranes. Membranes were then incubated with Hyb-75 buffer (20 mmol/L HEPES, pH 7.6, 75 mmol/L KCl, 0.1 mmol/L EDTA, 2.5 mmol/L MgCl2, 0.005% Nonidet P-40, 1 mmol/L DTT) supplemented with 5% non-fat milk, overnight at 4°C. Membranes were briefly washed with Hyb-75 buffer and then incubated with 1 µg/ml recombinant protein (PARP1 (Trevigen), pSmad3 (Cell Signaling), or β-actin (Abnova Corporation) respectively) at room temperature for 1 hour. After washed with Hyb-75 buffer, membranes were incubated with anti-PARP1 antibody (1∶1000) or anti-Smad3 (1∶800) antibody at 4°C overnight. After washing, membranes were incubated with HRP-conjugated secondary antibody for 2 hours. Specific bands were detected using the ECL detection system (Pierce).

### Immunoprecipitation assay

Immunoprecipitation assays were performed as described previously [41]. Briefly, 500 µg of nuclear extracts were incubated with the indicated antibodies (anti-PARP1, anti-Smad3, anti-PAR, or unspecific IgG respectively) at 4°C for 1 hour, and protein-G agarose at 4°C for 12 hours. The immunoprecipitates were pelleted by centrifugation at 5000 g for 1 minute and washed 4 times with lysis buffer. The pellets were suspended in SDS gel loading buffer, boiled for 10 minutes, and subjected to western blot analysis. To determine the specificity of the bands, unspecific IgG (negative control) were used.

### Electrophoretic Mobility Shift Assay (EMSA) and Supershift Assay

DNA-protein interaction was detected using LightShift™ Chemiluminescent EMSA kit (Pierce) according to the manufacturer's protocol. The sequence of SBE consensus oligonucleotides was: 5′-AGTATGTCTAGACTGA-3′. Biotin was labeled at the 5′end of the oligonucleotides. In supershift assay, after incubation of nuclear extracts with 2 µg of the indicated antibody or unspecific IgG at 4°C for 60 minutes, biotin-labeled oligonucleotides were added to the reaction and incubated for another 20 minutes.

### Southwestern Blot

Southwestern blot was performed according to the procedure of Butler and Ordahl [42] with slight modifications. Nuclear proteins (35 µg) were resolved on a 9% SDS-PAGE and then electrotransferred to a nitrocellulose membrane. Membranes were blocked with 5% BLOTTO–0.1% bovine serum albumin–1 mg/ml poly(dI–dC) in binding buffer (30 mM HEPES (pH 7.6), 1 mM dithiothreitol), followed by incubation with 1.0 pmol biotin-labeled SBE oligonucleotides in Hyb-50 buffer (30 mmol/L HEPES (pH7.6), 50 mmol/L KCl, 10 mmol/L MgCl2, 0.1 mmol/L EDTA, 1 mmol/L DTT, 5% BLOTTO, 0.1% bovine serum albumin, and 1 mg/ml poly(dI–dC)) at 4°C overnight. After washed three times (30 mmol/L HEPES (pH7.6), 50 mmol/L NaCl, 1% BLOTTO), membranes were incubated with streptavidin-horseradish peroxidase conjugate in blocking buffer (Pierce) for 15 min. Specific binding was detected with ECL detection reagents (Pierce) and band intensities were quantified as described above.

### Luciferase assay

A 0.6-kb promoter fragment (spanning from +866 bp to +1523 bp) from the rat TIMP1 gene was cloned by PCR from rat genomic DNA and inserted into the NheI/XhoI cut pGL3 basic luciferase expression vector (Promega) according to the procedure of El-Sayed Akool et al[22]. The complementary oligonucleotides used for pSBE2-luc (with two copies of SBE) construction were sence: 5′-TAAGTCTAGACGGCAGTCTAGAC-3′, and antisence: 5′-TCGAGTCTAGACTGCCGTCTAGACTTAGTAC-3′. The complementary oligonucleo- tides were inserted into the KpnI/Xho-I cut pGL3- promoter luciferase expression vector (Promega). VSMCs were transiently transfected by use of Lipofectamine 2000 (Invitrogen) in accordance with the manufacturer's instruction. After incubation for 24 hours, cells were harvested, lysed, and assayed for luciferase activity with Dual Luciferase Reporter Assay Kit (Promega) according to the manufacturer's instruction.

### Statistic analysis

Values are shown as mean±SEM of at least three independent experiments. The significance of differences was estimated by one-way ANOVA followed by Student-Newmann-Keuls multiple comparison tests. P<0.05 was considered significant. All statistical analyses were performed with SPSS software (version 11.0, SPSS Inc).

## Supporting Information

Figure S1 **Evaluation of siRNA transfection effiency in VSMCs.** A. Insets show a magnification (40T) of VSMCs transfected with Cy-3-labeled siRNA as indicated concentration (10, 20, 50 nmol/L, 48 h) by light microscopy (upper panel) and fluorescence microscopy (lower panel). B. VSMCs transfected with vehicle or Cy-3-labeled siRNA as indicated concentration (10, 20, 50 nmol/L, 48 h) were analyzed with flow cytometry. C, D, E. The effects of PARP1 siRNA (50 nmol/L, 48 h) (C), Smad3 siRNA (50 nmol/L, 48 h) (D) and Smad2 siRNA (50 nmol/L, 48 h) (E) on levels of PARP1, Smad3 and Smad2 were analyzed by western blot, respectively. Unrelated siRNA served as negative control. Data are expressed as the mean±S.E.M, n = 6. ## P<0.01 compared to the control group.
(TIF)

Figure S2 **Phosphorylated Smad3 (pSmad3) was poly(ADP-ribosy)lated by PARP1.** A. Far-western-blot assay was used to detect the binding of PARP1 to pSmad3 in VSMCs transfected with PARP1 or unrelated siRNA (50 nmol/L, 48 h). pSmad3 was used as probe. The effects of PARP1 siRNA on Smad3 levels were shown in lower panel. B. Immunoprecipitation of pSmad3-bound proteins from TGF-β1-treated VSMCs, followed by western-blot assay using anti-PARP1 antibody. Unspecific IgG served as negative control. C. Far-western-blot assay was used to detect the binding of PARP1 to pSmad3 in cell free system. D. Poly(ADP-ribosy)lation levels of 52 kD nuclear protein were assessed by western-blot assay using anti-PAR antibody. histone 1 served as loading control. E. Western-blot assay with anti-PAR Ab was used to detect the poly(ADP-ribosy)lation levels of pSmad3, Smad4 or p65 in cell-free system. Recombinant pSmad3, Smad4 or p65 protein was incubated with vehicle, PARP-1/NAD^+^/active DNA, PARP-1/NAD^+^/active DNA/3AB or PARP-1. Data are expressed as the mean±S.E.M, n = 6. ## P<0.01 compared to the control group. ** P<0.01 compared to the TGF-β1 treatment group.
(TIF)

Figure S3 **Poly(ADP-ribosy)lation increased DNA binding of Smad3 and Smad4.** A. EMSA assay of nuclear extracts from Smad2-knockdown (50 nmol/L, 48 h) or Smad3-knockdown (50 nmol/L, 48 h) VSMCs using SBE as probe. Cells were pretreated with vehicles (PBS) or 3AB (10 mmol/L, 24 h), followed by TGF-β1 (10 ng/ml, 2 h) or vehicles (PBS) treatment. B. Southwestern-blot assay was used to detect SBE binding of Smad3. VSMCs were treated as indicated. C. South-Western-blot assay was used to detect the DNA binding activity of Smad3, Smad4 or PPAR-γ in cell-free system. Recombinant pSmad3, Smad4 or PPAR-γ were incubated with vehicles, PARP1/NAD^+^/active DNA, PARP1/NAD^+^/active DNA/3AB or PARP1. D. EMSA assay using SBE as probe. Lane 1–2 indicated incubation of nuclear extracts with recombinant PARP1 protein (1∶40, 1∶20). Lane 4 indicated incubation of nuclear extracts with unspecific IgG. Lane 3 served as control. E. Southwestern-blot assay was used to detect SBE binding of Smad4. VSMCs were treated as indicated. F. Nuclear extracts were incubated with 3AB, NAD^+^/active DNA, or NAD^+^/active DNA/3AB. Southwestern-blot assay was used to detect SBE binding of Smad4. The effects of Smad2, Smad3 and Smad4 siRNA on levels of Smad2, Smad3 and Smad4 were shown in Figure S1. Data are expressed as the mean±S.E.M, n = 6. ## P<0.01 compared to the control group, ** P<0.01 compared to the NAD^+^ /active DNA treatment group.
(TIF)

Information S1 **Poly(ADP-ribosy)lation increased the DNA binding of Smad4**.
(DOC)

Table S1 **The sequences of siRNAs used in this study.**
(DOC)

Table S2 **The sequences of primers for real time RT-PCR used in this study.**
(DOC)

## References

[1] August P, Suthanthiran M (2006). Transforming growth factor beta signaling, vascular remodeling, and hypertension.. N Engl J Med.

[2] Khan R, Agrotis A, Bobik A (2007). Understanding the role of transforming growth factor-beta1 in intimal thickening after vascular injury.. Cardiovasc Res.

[3] Bobik A (2006). Transforming growth factor-betas and vascular disorders.. Arterioscler Thromb Vasc Biol.

[4] Tsai S, Hollenbeck ST, Ryer EJ, Edlin R, Yamanouchi D (2009). TGF-beta through Smad3 signaling stimulates vascular smooth muscle cell proliferation and neointimal formation.. Am J Physiol Heart Circ Physiol.

[5] Wang W, Huang XR, Canlas E, Oka K, Truong LD (2006). Essential role of Smad3 in angiotensin II-induced vascular fibrosis.. Circ Res.

[6] Ruiz-Ortega M, Rodriguez-Vita J, Sanchez-Lopez E, Carvajal G, Egido J (2007). TGF-beta signaling in vascular fibrosis.. Cardiovasc Res.

[7] Shi Y, Massague J (2003). Mechanisms of TGF-beta signaling from cell membrane to the nucleus.. Cell.

[8] Sturrock A, Cahill B, Norman K, Huecksteadt TP, Hill K (2006). Transforming growth factor-beta1 induces Nox4 NAD(P)H oxidase and reactive oxygen species-dependent proliferation in human pulmonary artery smooth muscle cells.. Am J Physiol Lung Cell Mol Physiol.

[9] Liu RM, Gaston Pravia KA (2010). Oxidative stress and glutathione in TGF-beta-mediated fibrogenesis.. Free Radic Biol Med.

[10] Huang D, Wang Y, Yang C, Liao Y, Huang K (2009). Angiotensin II promotes poly(ADP-ribosyl)ation of c-Jun/c-Fos in cardiac fibroblasts.. J Mol Cell Cardiol.

[11] Huang D, Yang C, Wang Y, Liao Y, Huang K (2009). PARP1 suppresses adiponectin expression through poly(ADP-ribosyl)ation of PPAR gamma in cardiac fibroblasts.. Cardiovasc Res.

[12] D'Amours D, Desnoyers S, D'Silva I, Poirier GG (1999). Poly(ADP-ribosyl)ation reactions in the regulation of nuclear functions.. Biochem J.

[13] Wiseman H, Halliwell B (1996). Damage to DNA by reactive oxygen and nitrogen species: role in inflammatory disease and progression to cancer.. Biochem J.

[14] Pillai JB, Gupta M, Rajamohan SB, Lang R, Raman J (2006). Poly(ADP-ribose) polymerase-1-deficient mice are protected from angiotensin II-induced cardiac hypertrophy.. Am J Physiol Heart Circ Physiol.

[15] Chiu J, Farhangkhoee H, Xu BY, Chen S, George B (2008). PARP mediates structural alterations in diabetic cardiomyopathy.. J Mol Cell Cardiol.

[16] Shevalye H, Maksimchyk Y, Watcho P, Obrosova IG (2010). Poly(ADP-ribose) polymerase-1 (PARP1) gene deficiency alleviates diabetic kidney disease.. Biochim Biophys Acta.

[17] Doran AC, Meller N, McNamara CA (2008). Role of smooth muscle cells in the initiation and early progression of atherosclerosis.. Arterioscler Thromb Vasc Biol.

[18] Soldatenkov VA, Chasovskikh S, Potaman VN, Trofimova I, Smulson ME (2002). Transcriptional repression by binding of poly(ADP-ribose) polymerase to promoter sequences.. J Biol Chem.

[19] Vidakovic M, Gluch A, Qiao J, Oumard A, Frisch M (2009). PARP1 expression in the mouse is controlled by an autoregulatory loop: PARP1 binding to an upstream S/MAR element and to a novel recognition motif in its promoter suppresses transcription.. J Mol Biol.

[20] Wrighton KH, Lin X, Feng XH (2009). Phospho-control of TGF-beta superfamily signaling.. Cell Res.

[21] Massague J (1998). TGF-beta signal transduction.. Annu Rev Biochem.

[22] Akool el-S, Doller A, Muller R, Gutwein P, Xin C (2005). Nitric oxide induces TIMP-1 expression by activating the transforming growth factor beta-Smad signaling pathway.. J Biol Chem.

[23] Cooke MS, Evans MD, Dizdaroglu M, Lunec J (2003). Oxidative DNA damage: mechanisms, mutation, and disease.. FASEB J.

[24] Hong YH, Peng HB, La Fata V, Liao JK (1997). Hydrogen peroxide-mediated transcriptional induction of macrophage colony-stimulating factor by TGF-beta1.. J Immunol.

[25] Jiang Z, Seo JY, Ha H, Lee EA, Kim YS (2003). Reactive oxygen species mediate TGF-beta1-induced plasminogen activator inhibitor-1 upregulation in mesangial cells.. Biochem Biophys Res Commun.

[26] Abdallah Y, Gligorievski D, Kasseckert SA, Dieterich L, Schafer M (2007). The role of poly(ADP-ribose) polymerase (PARP) in the autonomous proliferative response of endothelial cells to hypoxia.. Cardiovasc Res.

[27] Lonn P, van der Heide LP, Dahl M, Hellman U, Heldin CH (2010). PARP1 Attenuates Smad-Mediated Transcription.. Mol Cell.

[28] Pierreux CE, Nicolas FJ, Hill CS (2000). Transforming growth factor beta-independent shuttling of Smad4 between the cytoplasm and nucleus.. Mol Cell Biol.

[29] Olabisi OA, Soto-Nieves N, Nieves E, Yang TT, Yang X (2008). Regulation of transcription factor NFAT by ADP-ribosylation.. Mol Cell Biol.

[30] Grainger DJ (2007). TGF-beta and atherosclerosis in man.. Cardiovasc Res.

[31] Hill CS (2009). Nucleocytoplasmic shuttling of Smad proteins.. Cell Res.

[32] Xu L (2006). Regulation of Smad activities.. Biochim Biophys Acta.

[33] Lin X, Duan X, Liang YY, Su Y, Wrighton KH (2006). PPM1A functions as a Smad phosphatase to terminate TGFbeta signaling.. Cell.

[34] Dai F, Lin X, Chang C, Feng XH (2009). Nuclear export of Smad2 and Smad3 by RanBP3 facilitates termination of TGF-beta signaling.. Dev Cell.

[35] Kurisaki A, Kurisaki K, Kowanetz M, Sugino H, Yoneda Y (2006). The mechanism of nuclear export of Smad3 involves exportin 4 and Ran.. Mol Cell Biol.

[36] Varelas X, Sakuma R, Samavarchi-Tehrani P, Peerani R, Rao BM (2008). TAZ controls Smad nucleocytoplasmic shuttling and regulates human embryonic stem-cell self-renewal.. Nat Cell Biol.

[37] Liu RM, Liu Y, Forman HJ, Olman M, Tarpey MM (2004). Glutathione regulates transforming growth factor-beta-stimulated collagen production in fibroblasts.. Am J Physiol Lung Cell Mol Physiol.

[38] Sugiura H, Ichikawa T, Liu X, Kobayashi T, Wang XQ (2009). N-acetyl-L-cysteine inhibits TGF-beta1-induced profibrotic responses in fibroblasts.. Pulm Pharmacol Ther.

[39] Battle T, Arnal JF, Challah M, Michel JB (1994). Selective isolation of rat aortic wall layers and their cell types in culture–application to converting enzyme activity measurement.. Tissue Cell.

[40] Kitsera N, Khobta A, Epe B (2007). Destabilized green fluorescent protein detects rapid removal of transcription blocks after genotoxic exposure.. Biotechniques.

[41] Zeng C, Luo Y, Asico LD, Hopfer U, Eisner GM (2003). Perturbation of D1 dopamine and AT1 receptor interaction in spontaneously hypertensive rats.. Hypertension.

[42] Butler AJ, Ordahl CP (1999). Poly(ADP-ribose) polymerase binds with transcription enhancer factor 1 to MCAT1 elements to regulate muscle-specific transcription.. Mol Cell Biol.

