# Supplementary figures and images for: Poly(ADP-ribose) Polymerase 1 Is Indispensable for Transforming Growth Factor-β Induced Smad3 Activation in Vascular Smooth Muscle Cell
Source: PLoS One. 2011 Oct 31;6(10):e27123. doi: 10.1371/journal.pone.0027123 (PMC3205050; doi:10.1371/journal.pone.0027123)

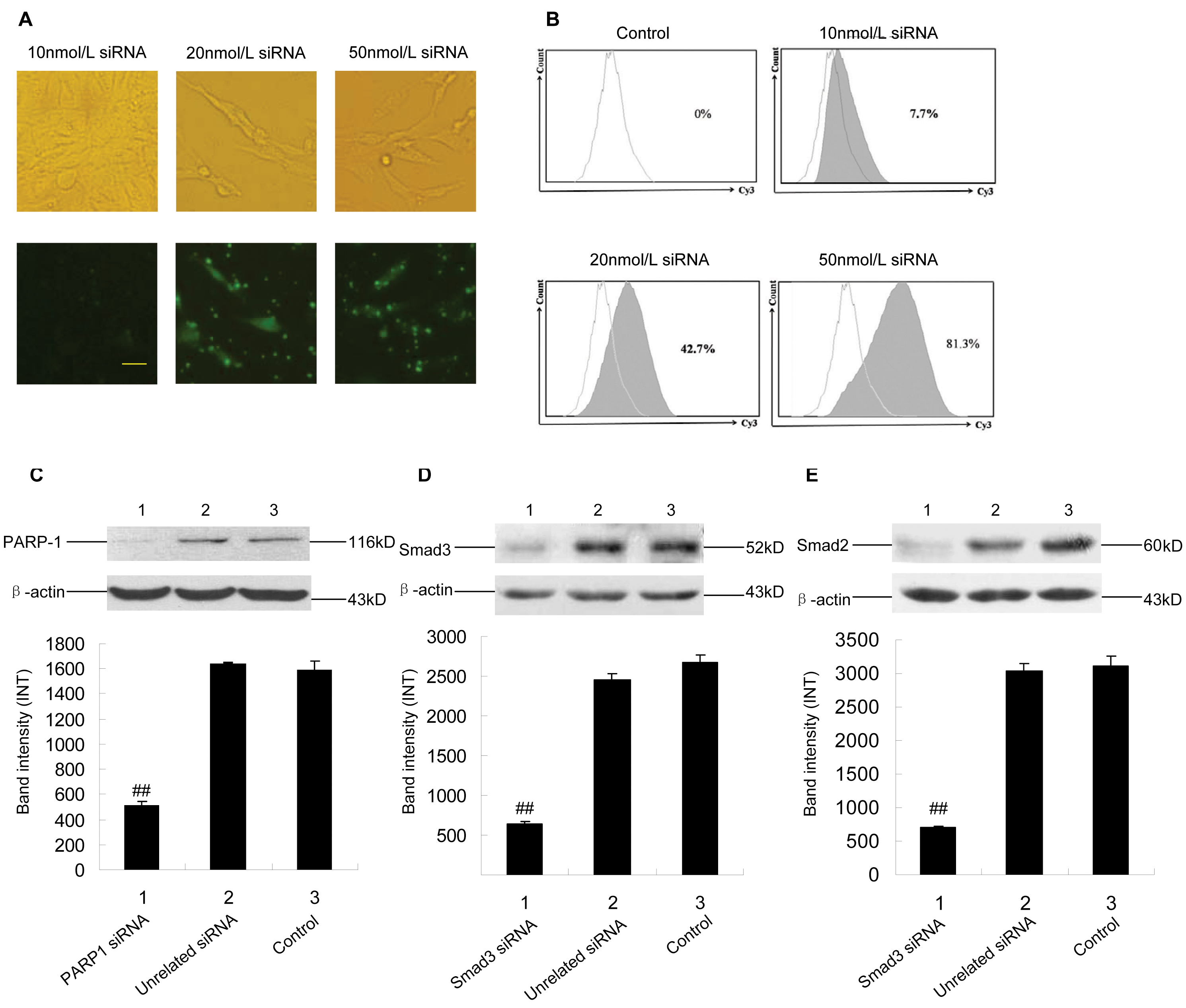

Supplement: Figure S1 — Evaluation of siRNA transfection effiency in VSMCs. A. Insets show a magnification (40T) of VSMCs transfected with Cy-3-labeled siRNA as indicated concentration (10, 20, 50 nmol/L, 48 h) by light microscopy (upper panel) and fluorescence microscopy (lower panel). B. VSMCs transfected with vehicle or Cy-3-labeled siRNA as indicated concentration (10, 20, 50 nmol/L, 48 h) were analyzed with flow cytometry. C, D, E. The effects of PARP1 siRNA (50 nmol/L, 48 h) (C), Smad3 siRNA (50 nmol/L, 48 h) (D) and Smad2 siRNA (50 nmol/L, 48 h) (E) on levels of PARP1, Smad3 and Smad2 were analyzed by western blot, respectively. Unrelated siRNA served as negative control. Data are expressed as the mean±S.E.M, n = 6. ## P<0.01 compared to the control group. (TIF) [file pone.0027123.s001.tif]

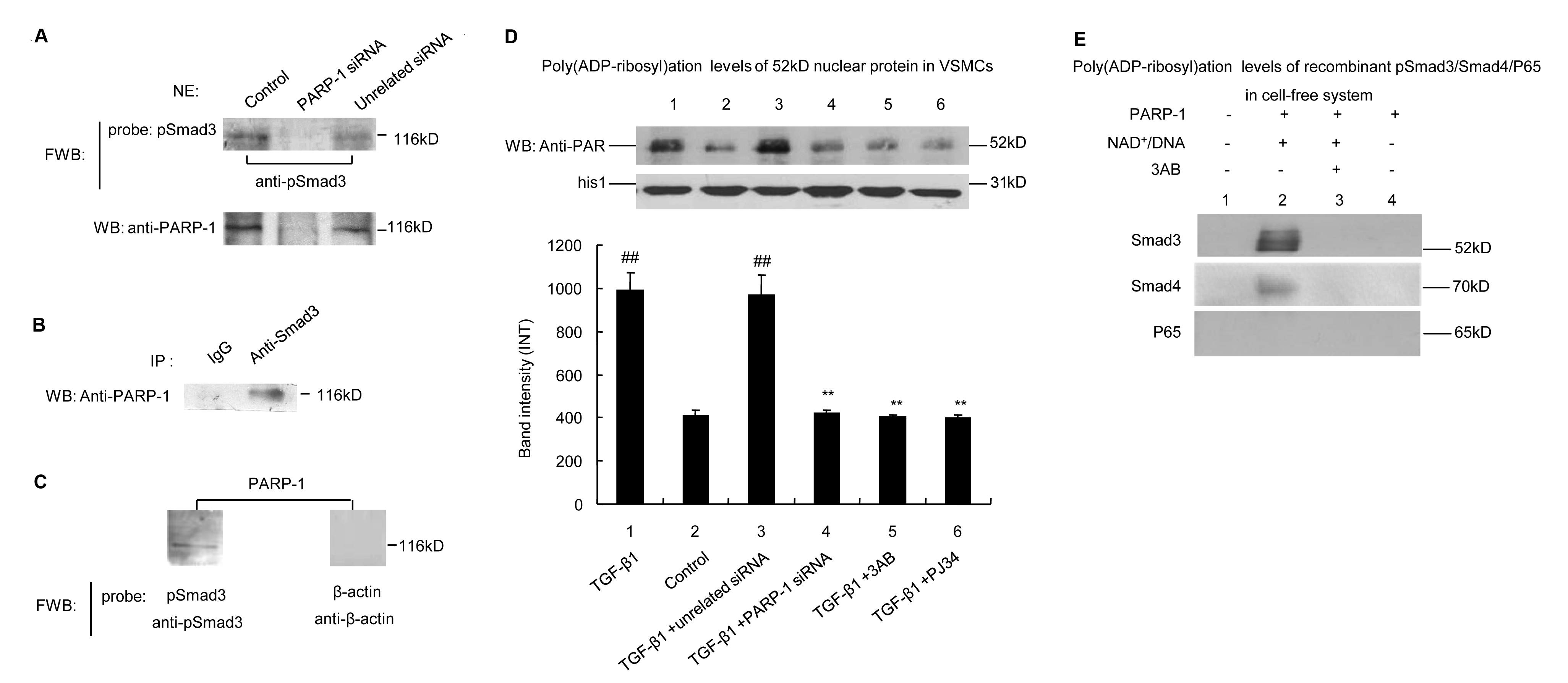

Supplement: Figure S2 — Phosphorylated Smad3 (pSmad3) was poly(ADP-ribosy)lated by PARP1. A. Far-western-blot assay was used to detect the binding of PARP1 to pSmad3 in VSMCs transfected with PARP1 or unrelated siRNA (50 nmol/L, 48 h). pSmad3 was used as probe. The effects of PARP1 siRNA on Smad3 levels were shown in lower panel. B. Immunoprecipitation of pSmad3-bound proteins from TGF-β1-treated VSMCs, followed by western-blot assay using anti-PARP1 antibody. Unspecific IgG served as negative control. C. Far-western-blot assay was used to detect the binding of PARP1 to pSmad3 in cell free system. D. Poly(ADP-ribosy)lation levels of 52 kD nuclear protein were assessed by western-blot assay using anti-PAR antibody. histone 1 served as loading control. E. Western-blot assay with anti-PAR Ab was used to detect the poly(ADP-ribosy)lation levels of pSmad3, Smad4 or p65 in cell-free system. Recombinant pSmad3, Smad4 or p65 protein was incubated with vehicle, PARP-1/NAD+/active DNA, PARP-1/NAD+/active DNA/3AB or PARP-1. Data are expressed as the mean±S.E.M, n = 6. ## P<0.01 compared to the control group. ** P<0.01 compared to the TGF-β1 treatment group. (TIF) [file pone.0027123.s002.tif]

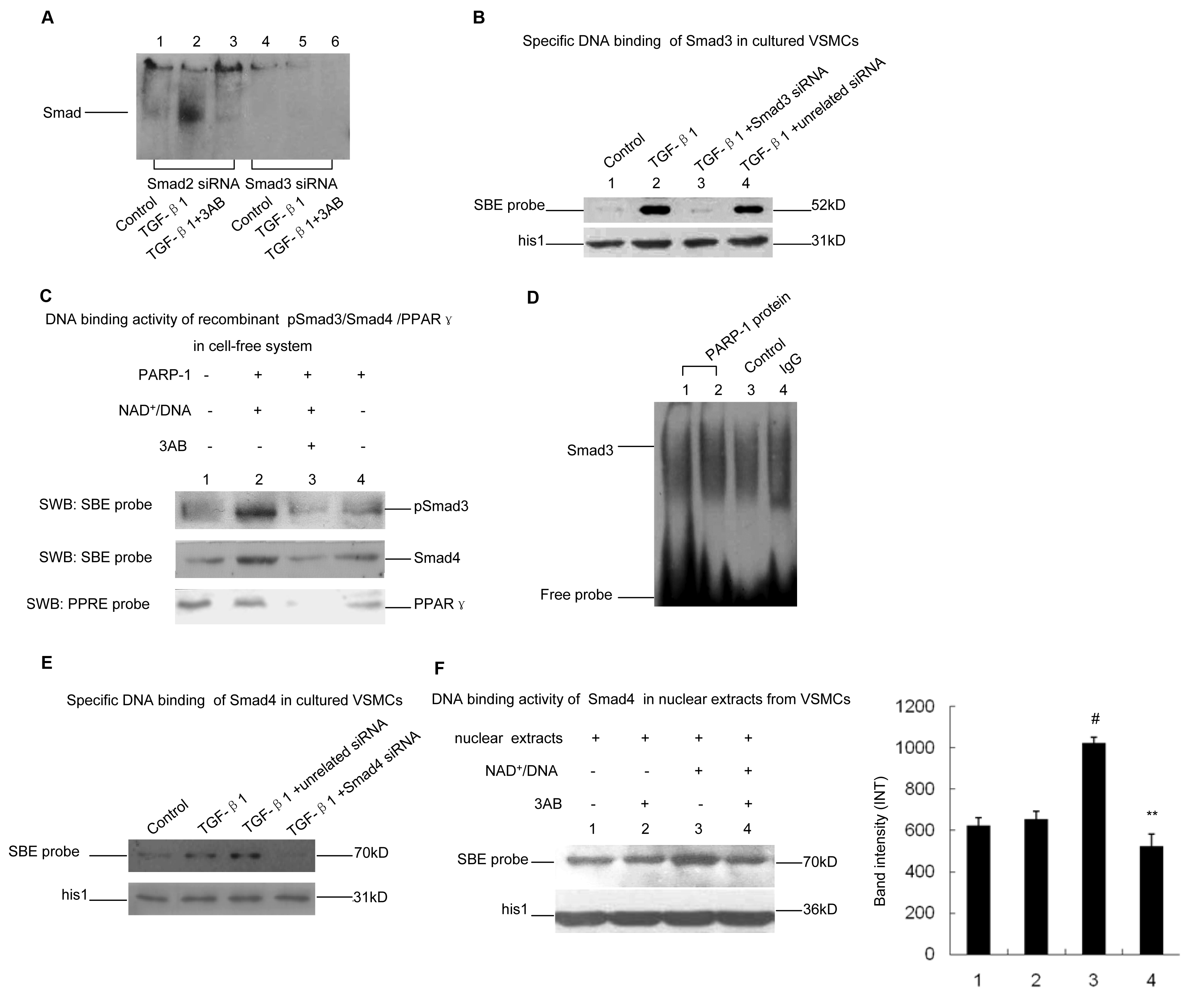

Supplement: Figure S3 — Poly(ADP-ribosy)lation increased DNA binding of Smad3 and Smad4. A. EMSA assay of nuclear extracts from Smad2-knockdown (50 nmol/L, 48 h) or Smad3-knockdown (50 nmol/L, 48 h) VSMCs using SBE as probe. Cells were pretreated with vehicles (PBS) or 3AB (10 mmol/L, 24 h), followed by TGF-β1 (10 ng/ml, 2 h) or vehicles (PBS) treatment. B. Southwestern-blot assay was used to detect SBE binding of Smad3. VSMCs were treated as indicated. C. South-Western-blot assay was used to detect the DNA binding activity of Smad3, Smad4 or PPAR-γ in cell-free system. Recombinant pSmad3, Smad4 or PPAR-γ were incubated with vehicles, PARP1/NAD+/active DNA, PARP1/NAD+/active DNA/3AB or PARP1. D. EMSA assay using SBE as probe. Lane 1–2 indicated incubation of nuclear extracts with recombinant PARP1 protein (1∶40, 1∶20). Lane 4 indicated incubation of nuclear extracts with unspecific IgG. Lane 3 served as control. E. Southwestern-blot assay was used to detect SBE binding of Smad4. VSMCs were treated as indicated. F. Nuclear extracts were incubated with 3AB, NAD+/active DNA, or NAD+/active DNA/3AB. Southwestern-blot assay was used to detect SBE binding of Smad4. The effects of Smad2, Smad3 and Smad4 siRNA on levels of Smad2, Smad3 and Smad4 were shown in Figure S1. Data are expressed as the mean±S.E.M, n = 6. ## P<0.01 compared to the control group, ** P<0.01 compared to the NAD+ /active DNA treatment group. (TIF) [file pone.0027123.s003.tif]
