# Supplementary material for: Poly(ADP-ribose) Polymerase 1 Is Indispensable for Transforming Growth Factor-β Induced Smad3 Activation in Vascular Smooth Muscle Cell
Source: PLoS One. 2011 Oct 31;6(10):e27123. doi: 10.1371/journal.pone.0027123 (PMC3205050; doi:10.1371/journal.pone.0027123)
Supplement: Information S1 — Poly(ADP-ribosy)lation increased the DNA binding of Smad4. (DOC) [file pone.0027123.s004.doc]

**Supporting Information**

**Supporting Information Results**

**Poly(ADP-ribosy)lation increased the DNA binding of Smad4**

Smad4 is a 70kD protein and distributes in both cytoplasm and nucleus of cells. Far-western-blot assay showed that auto-poly(ADP-ribosyl)ated PARP1 (AP-PARP1), but not UP-PARP1, could specifically bind to a 70kD nuclear protein in nontreated and TGF-β1-treated cells (Figure 3A). Additionally, TGF-β1 administration promoted poly(ADP-ribosy)lation of a 70kD nuclear protein in rat VSMCs (data not shown). In cell free system, incubation of Smad4 with PARP1, NAD^+^ and active DNA resulted in poly(ADP-ribosy)lation of Smad4 (Figure S2E).

The effect of poly(ADP-ribosy)lation on Smad4 DNA binding was explored. TGF-β1 treatment increased SBE binding of a 70kD nuclear protein; and knockdown of Smad4 by siRNA abrogated the binding (Figure S3E), indicating that the SBE-bound 70kD nuclear protein was Smad4. Incubation of nuclear extracts with PARP1, NAD^+^ and active DNA promoted binding of Smad4 to SBE, and the increase was inhibited by co-incubation with 3AB (Figure S4F). Similar results were found in cell free experiment (Figure S3C). All the data indicated that poly(ADP-ribosy)lation increased DNA binding of Smad4.

**Supporting Information Legends**

**Figure S1** Evaluation of siRNA transfection effiency in VSMCs.

A. Insets show a magniﬁcation (40T) of VSMCs transfected with Cy-3-labeled siRNA as indicated concentration (10, 20, 50nmol/L, 48h) by light microscopy (upper panel) and ﬂuorescence microscopy (lower panel). B. VSMCs transfected with vehicle or Cy-3-labeled siRNA as indicated concentration (10, 20, 50nmol/L, 48h) were analyzed with flow cytometry. C, D, E. The effects of PARP1 siRNA (50nmol/L, 48h) (C), Smad3 siRNA (50nmol/L, 48h) (D) and Smad2 siRNA (50nmol/L, 48h) (E) on levels of PARP1, Smad3 and Smad2 were analyzed by western blot, respectively. Unrelated siRNA served as negative control. Data are expressed as the mean±S.E.M, n=6. ## P<0.01 compared to the control group.

**Figure S2** Phosphorylated Smad3 (pSmad3) was poly(ADP-ribosy)lated by PARP1.

A. Far-western-blot assay was used to detect the binding of PARP1 to pSmad3 in VSMCs transfected with PARP1 or unrelated siRNA (50nmol/L, 48h). pSmad3 was used as probe. The effects of PARP1 siRNA on Smad3 levels were shown in lower panel. B. Immunoprecipitation of pSmad3-bound proteins from TGF-β1-treated VSMCs, followed by western-blot assay using anti-PARP1 antibody. Unspecific IgG served as negative control. C. Far-western-blot assay was used to detect the binding of PARP1 to pSmad3 in cell free system. D. Poly(ADP-ribosy)lation levels of 52kD nuclear protein were assessed by western-blot assay using anti-PAR antibody. histone 1 served as loading control. E. Western-blot assay with anti-PAR Ab was used to detect the poly(ADP-ribosy)lation levels of pSmad3, Smad4 or p65 in cell-free system. Recombinant pSmad3, Smad4 or p65 protein was incubated with vehicle, PARP-1/NAD^+^/active DNA, PARP-1/NAD^+^/active DNA/3AB or PARP-1. Data are expressed as the mean±S.E.M, n=6. ## P<0.01 compared to the control group. ** P<0.01 compared to the TGF-β1 treatment group.

**Figure S3** Poly(ADP-ribosy)lation increased DNA binding of Smad3 and Smad4.

A. EMSA assay of nuclear extracts from Smad2-knockdown (50nmol/L, 48h) or Smad3-knockdown (50nmol/L, 48h) VSMCs using SBE as probe. Cells were pretreated with vehicles (PBS) or 3AB (10mmol/L, 24h), followed by TGF-β1 (10ng/ml, 2h) or vehicles (PBS) treatment. B. Southwestern-blot assay was used to detect SBE binding of Smad3. VSMCs were treated as indicated. C. South-Western-blot assay was used to detect the DNA binding activity of Smad3, Smad4 or PPAR-γ in cell-free system. Recombinant pSmad3, Smad4 or PPAR-γ were incubated with vehicles, PARP1/NAD^+^/active DNA, PARP1/NAD^+^/active DNA/3AB or PARP1. D. EMSA assay using SBE as probe. Lane 1-2 indicated incubation of nuclear extracts with recombinant PARP1 protein (1:40, 1:20). Lane 4 indicated incubation of nuclear extracts with unspecific IgG. Lane 3 served as control. E. Southwestern-blot assay was used to detect SBE binding of Smad4. VSMCs were treated as indicated. F. Nuclear extracts were incubated with 3AB, NAD^+^/active DNA, or NAD^+^/active DNA/3AB. Southwestern-blot assay was used to detect SBE binding of Smad4. The effects of Smad2, Smad3 and Smad4 siRNA on levels of Smad2, Smad3 and Smad4 were shown in Supplemental Fig 1. Data are expressed as the mean±S.E.M, n=6. ## P<0.01 compared to the control group, ** P<0.01 compared to the NAD^+^ /active DNA treatment group.

**Table S1** The sequences of siRNAs used in this study.

**Table S2** The sequences of primers for real time RT-PCR used in this study.
