# Supplementary material for: Poly(ADP-ribose) Polymerase 1 Is Indispensable for Transforming Growth Factor-β Induced Smad3 Activation in Vascular Smooth Muscle Cell
Source: PLoS One. 2011 Oct 31;6(10):e27123. doi: 10.1371/journal.pone.0027123 (PMC3205050; doi:10.1371/journal.pone.0027123)
Supplement: Table S1 — The sequences of siRNAs used in this study. (DOC) [file pone.0027123.s005.doc]

**Table S1** The sequences of siRNAs used in this study

| siRNA | Sequence Sense (5'-3') |
| --- | --- |
| Rat PARP1 | 5'-GGAUGAUCUUCGACGUGGA-3' |
| Rat Smad2 | 5'-GCUGCCACAUGUUAUAUAU-3' |
| Rat Smad3 | 5'-UGGUGCGAGAAGGCGGUCA-3' |
| Unrelated siRNA | 5'-UUCUCCGAACGUGUCACGU-3' |
