# Supplementary material for: Poly(ADP-ribose) Polymerase 1 Is Indispensable for Transforming Growth Factor-β Induced Smad3 Activation in Vascular Smooth Muscle Cell
Source: PLoS One. 2011 Oct 31;6(10):e27123. doi: 10.1371/journal.pone.0027123 (PMC3205050; doi:10.1371/journal.pone.0027123)
Supplement: Table S2 — The sequences of primers for real time RT-PCR used in this study. (DOC) [file pone.0027123.s006.doc]

**Table S2 The sequences of primers for real time RT-PCR used in this study.**

| Gene | Primer sequence (Forward and Reverse) | Annealing (℃) |
| --- | --- | --- |
| Rat collagen I α1 | 5’-TCACCTACAGCACGCTTG-3’ | 58 |
|  | 5’-GGTCTGTTTCCAGGGTTG-3’ |  |
| Rat collagen IIIα1 | 5’-ATATCAAACACGCAAGGC-3’ | 58 |
|  | 5’-GATTAAAGCAAGAGGAACAC-3’ |  |
| Rat TIMP-1 | 5’-TCTGGCATCCTCTTGTTGCTAT-3’ | 58 |
|  | 5’-CCACAGCGTCGAATCCTT-3’ |  |
| Rat MMP-9 | 5’-TTCAAGGACGGTCGGTATT-3’ | 58 |
|  | 5’-CTCTGAGCCTAGACCCAACTTA-3’ |  |
| Rat MMP-2 | 5’-CTGATAACCTGGATGCAGTCGT-3’ | 58 |
|  | 5’-CCAGCCAGTCCGATTTGA-3’ |  |
| Rat GAPDH | 5’-ACCACAGTCCATGCCATCAC-3’ | 58 |
|  | 5’-TCCACCACCCTGTTGCTGTA-3’ |  |
